# Case Report: Sequential Chemotherapy and Immunotherapy Produce Sustained Response in Osteosarcoma With High Tumor Mutational Burden

**DOI:** 10.3389/fendo.2021.625226

**Published:** 2021-06-18

**Authors:** Shuier Zheng, Fenglin Wang, Jin Huang, Yan Zhou, Quanjun Yang, Guowei Qian, Chenliang Zhou, Daliu Min, Lele Song, Zan Shen

**Affiliations:** ^1^ Department of Oncology, Shanghai Jiao Tong University Affiliated Sixth People’s Hospital, Shanghai, China; ^2^ The Medical Division, HaploX Biotechnology, Shenzhen, China; ^3^ Department of Pathology, Shanghai Jiao Tong University Affiliated Sixth People’s Hospital, Shanghai, China; ^4^ Department of Pharmacy, Shanghai Jiao Tong University Affiliated Sixth People’s Hospital, Shanghai, China

**Keywords:** osteosarcoma, immunotherapy, chemotherapy, tumor mutational burden, whole-exome sequencing, sequencing

## Abstract

**Background:**

Immunotherapy has provided an effective method for the treatment of many cancers. However, its efficacy in osteosarcoma is not satisfactory so far.

**Case Presentation:**

Here, we presented a case of osteosarcoma treated with sequential chemotherapy and immunotherapy and showed promising therapeutic potential. The 29-year-old female patient presented 9^th^ rib osteosarcoma with suspected right lung lower lobe metastasis. Surgery was performed to remove the primary lesion, and a series of chemotherapies were given afterward in consideration of the response and tolerance. The right lung lower lobe metastasis was under control first but progressed (PD) 9 months after the initiation of therapy. The lesion was surgically removed and subsequent chemotherapy was implemented. The patient had good tolerance with chemotherapy and maintained well for approximately 11 months before the discovery of 11^th^ rib and right lung upper lobe metastases. Surgery was then performed on both lesions and achieved complete response. Post-surgical brief chemotherapy and subsequent long-term immunotherapy (pembrolizumab) maintained continuous remission for 33 months. The patient survived for 60 months with well-controlled disease from the time of confirmed diagnosis. Genetic alterations of all primary and metastatic lesions were investigated by whole-exome sequencing (WES). Substantial similarity in mutational landscape between the primary lesion and 11^th^ rib metastasis and between the two lung metastases were revealed, while substantial heterogeneity was found between the rib lesions and lung metastases. The tumor mutational burden (TMB) for the 9^th^ rib primary lesion, the metastatic 11^th^ rib lesion, and the metastatic right upper and lower lobe nodule tissues was 8.02, 2.38, 4.61, and 0.14 mutations/Mb, respectively. The primary lesion exhibited the most diverse copy number variation (CNV) changes among all lesions. Furthermore, pathway enrichment analysis also suggested significant heterogeneity among the lesions.

**Conclusions:**

Surgery with sequential chemotherapy and maintenance immunotherapy was shown to have good response for the first time on osteosarcoma patient who had high TMB tumor lesions and good tolerance for chemotherapy and immunotherapy.

## Introduction

Osteosarcoma is a bone malignancy originated from bone and rarely from soft tissue. They produce local invasion and often metastatic disease progression. Pulmonary metastasis is the most common metastases for osteosarcoma and over 90% of patients died from pulmonary metastases if untreated (1). The 5-year overall survival rate was 63% (95% confidence interval, 60%–66%) for all osteosarcoma patients and 71% (95% confidence interval, 67%–76%) for patients with non-metastatic disease (2). Patients with metastatic or recurrent disease had poor overall survival rate of less than 20% (3).

The conventional therapy for resectable osteosarcoma is surgery with adjuvant chemotherapy, while neoadjuvant chemotherapy, targeted therapy, and immunotherapy emerged in recent years as new options for advanced osteosarcoma. Based on recent advances in immunotherapy of osteosarcoma, the response rate with immune checkpoint inhibitors (ICIs), including pembrolizumab, nivolumab, ipilimumab, durvalumab, and avelumab, was not satisfactory from several reports with latest data (4). This suggests that ICIs alone may not provide enough efficacy for the treatment of osteosarcoma, and combined therapy may be one of the options to enhance efficacy. Here, we present a case of metastatic osteosarcoma treated with sequential chemotherapy and immunotherapy. The patients exhibited sustained response and good tolerance to chemotherapy and subsequent immunotherapy, and maintained responsive for 60 months. WES revealed high TMB for primary lesion and one of the metastatic lesion, which could be related to the good response. Our study provided the first piece of evidence for the effectiveness of sequential chemotherapy and immunotherapy for metastatic osteosarcoma.

## Case Presentation

**Figure 1:**
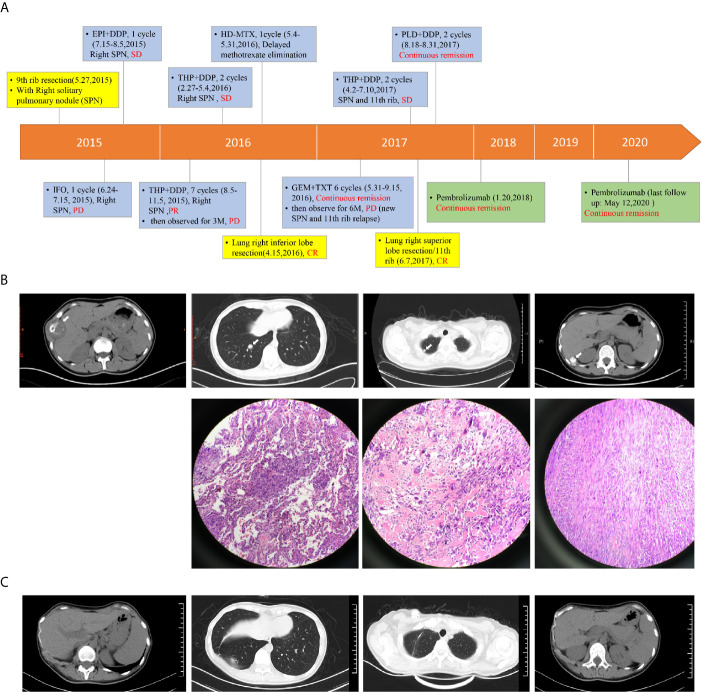
The therapeutic procedure and response of the patient. Panel **(A)** the flowchart shows the timeline of therapeutic regimes, duration of therapy and patient response. Types of therapies are highlighted in different colors. Blue: chemotherapy; Yellow: surgery; Green: immunotherapy. Panel **(B)** from left to right, the CT images show the primary cancer lesion at the 9^th^ rib, the right lower lobe metastasis, the right upper lobe metastasis, and the 11^th^ rib metastasis. Arrows indicate the lesions. The HE-stained images were also shown for the three metastases. The HE stained images for the primary cancer tissue was not available due to inaccessible biopsy. Panel **(C)** from left to right, the CT images show the status of right 9^th^ rib, right lung lower lobe, right lung upper lobe and right 11^th^ rib at May, 2020. EPI, epirubicin; DDP, cisplatin; SPN, solitary pulmonary nodule; THP, pirarubicin; HD-MTX, high-dose methotrexate; PLD, pegylated liposomal doxorubicin; IFO, ifosphamide; GEM, gemcitabine; TXT, taxotere. Details of chemotherapy and immunotherapy can be found in **Supplementary Table 1**.

Here we describe a 29-year-old female patient presenting 9^th^ rib bone destruction, peripheral tissue swelling and solitary pulmonary nodule (SPN) at the right lung lower lobe at the first visit in May 2015 (**Figures 1A, B** **)**. The lesion of the 9^th^ rib was removed by surgery and confirmed to be osteosarcoma by pathological examination. The SPN was therefore suspected to be a metastatic lesion. Post-surgical chemotherapy with ifosphamide (IFO) was performed for one cycle but the SPN appeared to progress (PD, July 2015). The subsequent epirubicin+cisplatin (EPI+DDP) therapy for one cycle controlled the SPN progression (SD, August 2015) but with side effects, and the chemotherapy regime was changed to pirarubicin + cisplatin (THP+DDP) and maintained for seven cycles, and achieved partial response (PR, November 2015) for the SPN. The chemotherapy was then stopped to observe the SPN status. Unfortunately, the SPN appeared to progress again (PD, February 2016) after 3 months. Surgery was then performed to remove the SPN at the right inferior lobe (CR, April 2016). Post-surgical chemotherapy with high-dose methotrexate (HD-MTX) was provided but the patient exhibited serious side effects (May 2016). The regime was then changed to gemcitabine+taxotere (GEM+TXT) and maintained for six cycles with continuous remission (PR, September 2016). Chemotherapy was then stopped, and no progression was observed until six months after the cease of therapy (PD, Mar, 2017). A new SPN at the right superior lobe and a suspected relapse at the right 11th rib were observed (**Figure 1B**). THP+DDP was performed for two cycles before the SPN at the right superior lobe was surgically removed (CR, June, 2017). Pegylated liposomal doxorubicin (PLD)+DDP was used for two cycles after surgery and continuous remission was observed with the 11^th^ rib relapse (PR, Aug, 2017), however, the patient complaint about intolerable side effects. Immunotherapy with pembrolizumab (100 mg q3w) was therefore started (January 2018), and the patient remained with continuous remission until the last follow-up in May 2020 (**Figure 1C**). Details of regimes of chemotherapy and immunotherapy are provided in **Supplementary Table 1**.

**Figure 2:**
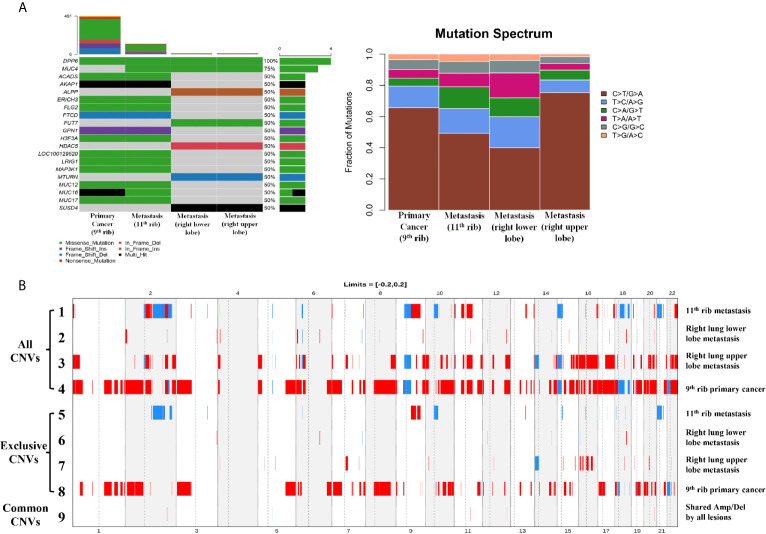
The mutational and copy number alterations for primary and metastatic lesions. The left figure of panel **(A)** shows the SNV/INDEL mutations of all lesions, and compared the common and exclusive mutations. The right figure of panel **(A)** shows the distribution and ratio of six types of base changes in the primary and metastatic lesions. Panel **(B)** shows the full profile of CNV alterations for all lesions (row 1–4) and exclusive CNV lesions for all lesions (row 5–8). Row 9 shows the common Amp/Del alterations from all lesions.

Whole-exome sequencing was performed with the 9^th^ rib primary lesion, the metastatic 11^th^ rib lesion, and the metastatic right upper and lower lobe nodule tissues. It can be observed from **Figure 2A** that a couple of mutations overlapped across the lesions, including DPP6 and MUC4, while large amount of mutations did not overlap between the rib lesions and the lung lesions. However, substantial overlaps of mutations were observed among the two rib lesions or the two lung lesions. Analysis of base change distribution shows big difference across the lesions (**Figure 2A**), especially the C>T/T>A base change (brown), which appeared to vary greatly across the four lesions. The tumor mutational burden (TMB) for the 9^th^ rib primary lesion, the metastatic 11^th^ rib lesion, and the metastatic right upper and lower lobe nodule tissues was 8.02, 2.38, 4.61, and 0.14 mutations/Mb, respectively. The driver genes of the lesions were determined by using five driver gene databases, including the CGC (5), IntOGen (6), BertVogelstein125 (7), SMG127 (8), and Comprehensive435 (9). **Table 1** shows that the 9^th^ rib primary lesion had large amount of driver genes, while few driver genes were found in other lesions. MAP3K1 and H3F3A were the only overlapped potential driver genes.

**Table 1:** Determination of driver gene mutations of all lesions of the patient by database interpretation.

	Gene name	Number of databases supporting driver gene	Primary cancer (9th rib)					Metastasis (11th rib)					Metastasis (right lung upper lobe)					Metastasis (right lung lower lobe)				
			CGC	IntOGen	BertVogelstein125	SMG127	Comprehensive435	CGC	IntOGen	BertVogelstein125	SMG127	Comprehensive435	CGC	IntOGen	BertVogelstein125	SMG127	Comprehensive435	CGC	IntOGen	BertVogelstein125	SMG127	Comprehensive435
SNV	MAP3K1	5	**√**	**√**	**√**	**√**	**√**	**√**	**√**	**√**	**√**	**√**	**√**	**√**	**√**	**√**	**√**	**-**	**-**	**-**	**-**	**-**
	NUP98	3	**√**	**√**	**-**	**-**	**√**	**-**	**-**	**-**	**-**	**-**	**-**	**-**	**-**	**-**	**-**	**-**	**-**	**-**	**-**	**-**
	POLE	2	**√**	**-**	**-**	**-**	**√**	**-**	**-**	**-**	**-**	**-**	**-**	**-**	**-**	**-**	**-**	**-**	**-**	**-**	**-**	**-**
	FAT1	2	**√**	**√**	**-**	**-**	**-**	**-**	**-**	**-**	**-**	**-**	**-**	**-**	**-**	**-**	**-**	**-**	**-**	**-**	**-**	**-**
	GNAQ	2	**√**	**-**	**√**	**-**	**-**	**-**	**-**	**-**	**-**	**-**	**-**	**-**	**-**	**-**	**-**	**-**	**-**	**-**	**-**	**-**
	H3F3A	2	**√**	**-**	**√**	**-**	**-**	**√**	**-**	**√**	**-**	**-**	**√**	**-**	**√**	**-**	**-**	**-**	**-**	**-**	**-**	**-**
	KMT2A	2	**√**	**-**	**-**	**-**	**√**	**-**	**-**	**-**	**-**	**-**	**-**	**-**	**-**	**-**	**-**	**-**	**-**	**-**	**-**	**-**
	USP9X	2	**-**	**-**	**-**	**√**	**√**	**-**	**-**	**-**	**-**	**-**	**-**	**-**	**-**	**-**	**-**	**-**	**-**	**-**	**-**	**-**
	RASA1	2	**-**	**√**	**-**	**-**	**√**	**-**	**-**	**-**	**-**	**-**	**-**	**-**	**-**	**-**	**-**	**-**	**-**	**-**	**-**	**-**
	NAV3	2	**-**	**-**	**-**	**√**	**√**	**-**	**-**	**-**	**-**	**-**	**-**	**-**	**-**	**-**	**-**	**-**	**-**	**-**	**-**	**-**
	GNAQ	2	**√**	**-**	**√**	**-**	**-**	**-**	**-**	**-**	**-**	**-**	**-**	**-**	**-**	**-**	**-**	**-**	**-**	**-**	**-**	**-**
INDEL	KDM6A	5	**√**	**√**	**√**	**√**	**√**	**-**	**-**	**-**	**-**	**-**	**-**	**-**	**-**	**-**	**-**	**-**	**-**	**-**	**-**	**-**
	PTEN	5	**√**	**√**	**√**	**√**	**√**	**-**	**-**	**-**	**-**	**-**	**-**	**-**	**-**	**-**	**-**	**-**	**-**	**-**	**-**	**-**
	GNAS	4	**√**	**√**	**√**	**-**	**√**	**-**	**-**	**-**	**-**	**-**	**-**	**-**	**-**	**-**	**-**	**-**	**-**	**-**	**-**	**-**
	ERBB2	4	**√**	**√**	**√**	**-**	**√**	**-**	**-**	**-**	**-**	**-**	**-**	**-**	**-**	**-**	**-**	**-**	**-**	**-**	**-**	**-**
	ERBB2	4	**√**	**√**	**√**	**-**	**√**	**-**	**-**	**-**	**-**	**-**	**-**	**-**	**-**	**-**	**-**	**-**	**-**	**-**	**-**	**-**
	GNAS	4	**√**	**√**	**√**	**-**	**√**	**-**	**-**	**-**	**-**	**-**	**-**	**-**	**-**	**-**	**-**	**-**	**-**	**-**	**-**	**-**

**Figure 3:**
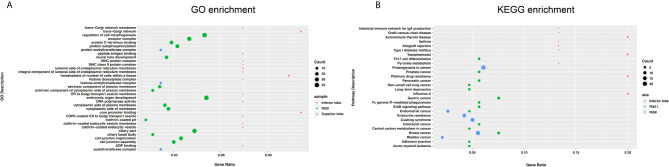
The summary of GO **(A)** and KEGG **(B)** enrichment. Primary and metastatic lesions with significant enrichment were shown. Significant variation in enrichment was observed among the lesions.

The CNV changes also showed substantial variation (**Figure 2B**). The 9^th^ rib primary cancer tissue appeared to have the maximal CNV changes (Line 4), followed by the right lung upper lobe metastasis (Line 3), while the right lung lower lobe (Line 2) and 11^th^ rib (Line 1) metastases appeared to have less CNVs. It is interesting to find that substantial amount of exclusive CNVs was observed in each lesion (Lines 5–8), especially for the 9^th^ rib primary cancer, which had large amount of exclusive CNVs (Line 8). However, not many common CNVs (Line 9) were found across the four lesions, and the chromosomes and genes with overlapped CNVs are shown in **Table 2**. The pathway enrichment analysis showed similar trend. The majority of GO enrichment was found in the 9^th^ rib primary lesion and the right lung inferior lobe metastasis, which had very little overlap in enrichment, while the other two lesions showed few or no enrichment (**Figure 3A**). In contrast, KEGG enrichment was found in the 9^th^ rib primary lesion, the 11^th^ rib metastasis, and the right lung inferior lobe metastasis, which also had little overlap in enrichment, while no significant enrichment was found with the right lung superior lobe metastasis (**Figure 3B**).

**Table 2:** Details of common Amp/Del alterations among four lesions.

Chromosome location	Alteration position (start and end position)		Type of CNV	Involved genes
11q14.1	78,000,000	78,950,000	Gain	GAB2,NARS2,TENM4
12q24.22-q24.21	116,500,000	117000000	Gain	MED13L
13q34	111,550,000	112250000	Gain	ANKRD10,ARHGEF7,TEX29
2q33.1	200,250,000	200550000	Gain	SATB2
20q13.2	51,850,000	52200000	Gain	TSHZ2,ZNF217
5q13.2	69,200,000	69550000	Loss	SERF1A,SERF1B,SMN1,SMN2
Xp11.23	49,200,000	49350000	Loss	GAGE12B,GAGE12C,GAGE12D,GAGE12E,GAGE12F,GAGE12G,GAGE12H,GAGE12I,GAGE12J,GAGE13,GAGE2A,GAGE2B,GAGE2C,GAGE2D,GAGE2E,GAGE4,GAGE5,GAGE6,GAGE7,GAGE8

## Discussion

Osteosarcoma patients with distant metastasis generally have worse prognosis than those without metastasis due to the invasive and metastatic nature of the disease (10). Surgery and post-surgical adjuvant chemotherapy have been the standard therapy for osteosarcoma patient with resectable lesions, while systematic therapies, including chemotherapy, targeted therapy, and immunotherapy, have been used for those with unresectable or metastatic lesions (10, 11). Immunotherapy has been suggested as a breakthrough for cancer treatment in recent years; however, the response of metastatic osteosarcoma to immunotherapy appeared to be limited with an objective response rate (ORR) of less than 20% from a series of recent clinical trials (4). Although PD-L1 expression rate was not low in osteosarcoma (4), pooled results showed that PD-L1/PD-1 overexpression was significantly associated with metastasis (RR = 1.54; 95% CI: 1.12–2.11, p = 0.008) in osteosarcoma, and patients exhibited a remarkably higher total mortality risk (RR = 1.86, 95% CI: 1.09–3.17, p = 0.021) with PD-L1/PD-1 overexpression (12). Therefore, PD-L1 expression may be an indicator for bad prognosis in osteosarcoma. Although patients with positive PD-1 expression generally exhibited better response than those with negative expression in solid tumors, metastasis may undermine the response to PD-1 drugs. This possibly explains the unsatisfactory response of PD-1 blockade to osteosarcoma compared with other solid tumors. Although combined therapy involving chemotherapy, targeted therapy, and immunotherapy has shown promising perspectives, more evidence is still needed to confirm the therapeutic response (4).

Apart from single and combined therapies, the sequential use of chemotherapy, radiotherapy, targeted therapy followed by immunotherapy has been emerging as one of the latest choices in cancer treatment and proved to be more effective than immunotherapy alone by some previous study in lung cancer, colorectal cancer, hepatocellular carcinoma, and cholangiocarcinoma (13–17). However, the sequential use of chemotherapy and immunotherapy in osteosarcoma has never been reported, and only *in vitro* evidence became available very recently, showing that checkpoint blockade in combination with doxorubicin augments tumor cell apoptosis (18), and neoadjuvant chemotherapy reprogrammed the tumor immunologic microenvironment and facilitated the subsequent immunotherapy in osteosarcoma (19). These evidence suggested that similar to the roles of chemotherapy prior to immunotherapy in the combined therapy of other cancers, treatment induced by chemotherapy and followed by immunotherapy may enhance the response to immunotherapy. Here, in this study, we provided the first clinical observation that patient could benefit from chemotherapy induction and subsequent immunotherapy. The strikingly longer survival in this patient compared with previous reports suggested that this regime might work in other patients with similar disease status.

One interesting observation is that pembrolizumab achieved more than 2 years continuous remission following the last surgery, in which the right upper metastasis was removed. The 11th rib metastasis also remained in control for more than 2 years. Patients entered into immunotherapy with a well-controlled status and maintained the status for a long time. In contrast, previous chemotherapy did not achieve this response, since two lung metastases progressed following chemotherapy. This observation suggested that maintenance therapy with pembrolizumab in this osteosarcoma patient with a well-controlled status could be a good option to prevent tumor recurrence. Sequential treatment with chemotherapy, surgery and immunotherapy with good timing may be crucial for lesion control. Furthermore, choice of reagents in chemotherapy might also affect the response in combined therapy with ICIs, since evidence suggested that Pirarubicin-based chemotherapy displayed better clinical outcomes and lower toxicity than doxorubicin-based chemotherapy in osteosarcoma treatment (20). It may be possible that anthracycline drugs exhibit better cancer control and lower toxicity than other types of chemotherapy drugs in osteosarcoma, especially in combination with ICIs in a sequential regime, but further investigation is needed to confirm the speculation.

One of the significant features of the patient was the high TMB revealed by WES in primary cancer tissue. The patient exhibited a TMB of 8.02 for the 9^th^ rib primary cancer and a TMB of 4.61 for the right upper lobe metastasis, which was way above the range of reported TMB in osteosarcoma. One study reported a median of 1.525 (25% and 75% percentile at 1.09 and 2.36 mutations/Mb) from 63 osteosarcoma patients (21). Another study reported a median of 2.5 from 283 osteosarcoma patients (22). Therefore, the TMB from the present case can safely be defined as high TMB based on the previous reports. Previous studies on the correlation between TMB and immunotherapy strongly supported the conclusion that high tumor mutation burden predicts better efficacy of immunotherapy and is an independent predictor of the response in immunotherapy (23, 24). Furthermore, some evidence suggested that TMB itself is a predictor of patient survival, regardless of therapeutic methods (25–27). This could also be true in osteosarcoma but evidence on the specific cancer type is still needed. Here we speculate that the extremely good response from this patient may be related to her high TMB and good tolerance of chemotherapy, which rendered the long-term use of various types of chemotherapy. The use of chemotherapy may facilitate the adaption to immunotherapy, and produced good response and tolerance to immunotherapy, especially in the background of high TMB. However, it is difficult to find appropriate therapeutic strategies for patients with low tolerance, as there are not many therapeutic options for osteosarcoma at the moment. It is a routine to start with standard therapy and choose more radical therapies if progression or resistance happens. There is currently no guideline for combined therapy, and doctors can only set up personalized regimes for certain patients based on patient individual responses and their experiences.

In this study, the patient exhibited distinct mutational landscape across primary cancer tissue and metastatic tissues. Substantial intertumoral heterogeneity was observed across the lesions. The primary lesion at 9^th^ rib and the 11^th^ rib metastasis appeared to have similar mutational landscape, while the other two lung metastatic lesions appeared to share similar mutations. This suggests that the 11^th^ rib metastasis could be local dissemination from the primary lesion, while the two lung lesions could be evolved from the same subclones of the primary lesion. However, the CNV change exhibited different heterogeneity, in which the primary lesion and the right lung upper lobe lesion appeared to be more similar to each other than any other pairs, while only a few common CNV segments were observed across the lesions. It can be suggested from these observations that the primary lesion had the most diverse alterations (SNV/INDEL and CNVs) than other lesions, which was the clonal origin of other metastases. Meanwhile, GO and KEGG pathway enrichment analyses also suggested that the aberrant pathway or significant altered functions exhibited significant heterogeneity, since little overlap was observed across the lesions.

It would be interesting to do *in vitro* experiments to examine the sensitivity of osteosarcoma to chemotherapy or immunotherapy before therapy using primary cell culture with tumor tissues from patients, or establish organoid models for drug selection. The main purposes for these experiments include the identification of the optimal single reagents and combinations for certain patient for imminent therapy. Fast results from drug sensitivity test would greatly help doctors choose the best therapy for patients. *In vivo* studies may involve clinical trials on osteosarcoma patients. Patients with high TMB and good tolerance may be the population benefit from chemotherapy combined with immunotherapy, but these patients account for only a small proportion in osteosarcoma. The rest large majority of patients may need combination of chemotherapy, radiotherapy, immunotherapy, and surgery. Doctors may have to establish personalized therapeutic strategies for different patients. Clinical trials may be started when certain combinations show a trend of significantly better response than others.

In conclusion, we revealed that sequential chemotherapy and immunotherapy may be a good choice for osteosarcoma patients who have high TMB tumor lesions, especially when patients have good tolerance for chemotherapy involving DDP and immunotherapy. Whether this is also true for patients with medium or low TMB still needs further investigation.

## Ethics Statement

The studies involving human participants were reviewed and approved by the Shanghai Sixth People’s Hospital Ethics Committee. The patients/participants provided their written informed consent to participate in this study. Written informed consent was obtained from the individual(s) for the publication of any potentially identifiable images or data included in this article.

## Author Contributions

SZ, LS, and ZS designed the study and were responsible for project management and implementation. SZ, JH, YZ, GQ, CZ, and DM were responsible for patient recruitment, sample collection, sample storage, transportation, and clinical information collection. FW and LS performed the sequencing experiments, collected the data, and performed the data analysis and initial interpretation. SZ, FW, and LS performed the final statistics and made the figures and tables. SZ, FW, and LS wrote the manuscript. SZ, LS, and ZS proofread the manuscript. LS submitted the manuscript. All authors contributed to the article and approved the submitted version.

## Funding

This study was supported by the National Natural Science Foundation of China (grant numbers: 81001192, 81672658, and 81972521) and National Key Research Project of Science and Technology Ministry (grant number: 2016YFC0106204).

## Conflict of Interest

FW and LS are employees of HaploX Biotechnology, who performed the NGS sequencing and data analysis in this study.

The remaining authors declare that the research was conducted in the absence of any commercial or financial relationships that could be construed as a potential conflict of interest.
