# Supplementary material for: Case Report: Sequential Chemotherapy and Immunotherapy Produce Sustained Response in Osteosarcoma With High Tumor Mutational Burden
Source: Front Endocrinol (Lausanne). 2021 Jun 18;12:625226. doi: 10.3389/fendo.2021.625226 (PMC8249865; doi:10.3389/fendo.2021.625226)
Supplement: Supplementary file 2 [file DataSheet_2.docx]

**Methods and materials**

*Ethic approval by participating hospitals*

All experiment plans and protocols for the study were submitted to the ethics/licensing committees of the named participating hospital for review and approval before the start of the clinical study, and were approved by the corresponding committees of the participating hospital. All experiments, methods, procedures and personnel training were carried out in accordance with relevant guidelines and regulations of the participating hospital and laboratories.

*Patients and samples*

Informed consent was obtained from the patient, who was informed about the test results. FFPE samples of primary cancer and metastases were collected from department of pathology of the hospital. The details of patient demographic and clinicopathological information were also collected. Diagnosis of cancer and metastases was confirmed by clinical assessments, imaging examinations (including CT and/or MRI) and pathological examinations.

*DNA extraction and quantification*

For the FFPE samples, ten 5 μm tissue sections were taken for DNA extraction, using the QIAamp DNA FFPE Kit (QIAGEN, Valencia, CA, USA), and following the manufacturer’s instructions. RelaxGene blood DNA system (Tiangen Biotech) was used to extract genomic DNA from peripheral blood lymphocytes (PBLs). The quality control for the DNA was achieved using Qubit 2.0 (Thermo Fisher Scientific), following the manufacturer’s instructions.

*Library construction, whole-exome sequencing, and data processing*

The fragmented genomic DNA underwent end-repairing, A-tailing and ligation, and then was sequentially completed with indexed adapters, followed by size selection using Agencourt AMPure XP beads (Beckman Coulter Inc., Brea, CA, USA). The DNA fragments were used for library construction with the KAPA Library Preparation kit (Kapa Biosystems, Inc., Wilmington, MA, USA) according to the manufacturer's protocol. Seven to eight polymerase chain reaction (PCR) cycles, depending on the amount of DNA used, were performed on pre-capture ligation-mediated PCR (Pre‑LM‑PCR) Oligos (Kapa Biosystems, Inc.) in 50μl reactions. The DNA sequencing was performed using a WESPlus gene panel (an upgraded version of the standard whole-exome sequencing (WES), HaploX Biotechnology) for tumor tissue sequencing on the Illumina Novaseq 6000 system according to the manufacturer's instructions.

Sequencing data were filtered by fastp and aligned to the hg19 genome (GRch37) using Burrows Wheeler Aligner (BWA). SAMtools was used to sort the BAM files and perform duplicate marking. The Gencore version 0.12.0 (https://github.com/OpenGene/gencore) was used to remove duplicate reads. Somatic variants were determined using MuTect2. New panel of normal (PON) created by in-house healthy individual using GATK. ANNOVAR was performed to annotate the Variant Call Format file obtained in the previous step. Data was visualized using the R software (<https://www.r-project.org/>) package ggplot2 (a model from the R software). Mutational landscape, clustering and enrichment analysis were performed with relevant packages of the R software.

The tumor mutation burden (TMB) was identified as the total number of incorrect codings, base substitutions, and insertions and deletions in somatic cells per one million bases. The TMB was calculated by dividing the total number of tissue non-synonymous SNV and INDEL variations (with allele frequencies ≥ 2%) by the size of the coding region covered by the WESPlus panel.
